# The diagnostic and prognostic significance of small nuclear ribonucleoprotein Sm D1 aberrantly high expression in hepatocellular carcinoma

**DOI:** 10.7150/jca.65225

**Published:** 2022-01-01

**Authors:** Huaxiang Wang, Fengfeng Xu, Lingling Lu, Fang Yang, Xinghua Huang, Lizhi Lv, Huanzhang Hu, Yi Jiang

**Affiliations:** 1 The Fuzong Clinical Medical College of Fujian Medical University, Fuzhou, Fujian 350025, PR China.; 2 Department of Hepatobiliary Surgery, 900 Hospital of the Joint Logistic Team, Fuzhou, Fujian 350025, PR China.

**Keywords:** SNRPD1, miR-100, Hepatocellular carcinoma, prognosis, mechanism.

## Abstract

Small nuclear ribonucleoprotein Sm D1 (SNRPD1), one of the crucial genes encoding core spliceosome components, was abnormally highly expressed in multiple types of tumors. In this study, we investigated the diagnostic and prognostic significance of SNRPD1 in hepatocellular carcinoma (HCC). The investigation of datasets from GEO and TCGA databases revealed that SNRPD1 expression in HCC was significantly higher than adjacent normal liver tissues, which was validated by immunohistochemistry (IHC). Both GO, KEGG analysis showed that the SNRPD1 co-expressed genes mainly enriched in Cell division, Nuclear import, mRNA splicing via spliceosome, Ribosome, Cell cycle, etc. Survival analysis from the GSE14520 dataset and 154 HCC cohorts exhibited a significant association of high SNRPD1 expression with poor overall survival and recurrence-free survival. ROC analysis showed that the abnormally high SNRPD1 mRNA expression has diagnostic significance in distinguishing between HCC and normal liver tissue (AUC = 0.819). Gene set enrichment analysis (GSEA) demonstrated that the high expression of SNRPD1 might regulate HCC tumorigenesis and progression by affecting the cell cycle, mismatch repair, DNA replication, and RNA degradation, etc. The luciferase report assay revealed that SNRPD1 was the direct target gene of miR-100 manifested by decreased SNRPD1 expression and luciferase activity in the HCC cells upon miR-100 overexpression. Finally, SNRPD1 may as an oncogene affecting the progression of HCC through regulates the mTOR pathway and autophagy.

## 1. Introduction

Hepatocellular carcinoma (HCC) causes a huge health-related economic burden globally, especially in East Asia and sub-Saharan Africa 1. Chronic hepatitis virus infections such as hepatitis B virus (HBV) and hepatitis C virus (HCV), long-term exposure to aflatoxins and chemical substances, alcohol consumption, and non-alcoholic fatty liver disease are high-risk factors for HCC 1, 2. Serum alpha-fetoprotein (AFP), the golden standard prognostic biomarker currently of HCC patients, has a low sensitivity and specificity due to was easily influenced by none-HCC disease. Therefore, in-depth investigation of the molecular mechanism and signaling pathways of the occurrence and development of HCC and the search for highly sensitive biomarkers are of great significance for the early diagnosis and prognosis of HCC.

Spliceosome, a dynamic macromolecular complex composed of five small nuclear ribonucleoproteins (termed U1, U2, U4, U5, and U6) and a set of proteins associated with spliceosome, was responsible for participating in the splicing of the pre-messenger RNA, such as removing introns and then the connection of exons in a certain order 3-5. Each small nuclear ribonucleoprotein was composed of small nuclear RNA and a set of 7 SNRP proteins (B/B', D1, D2, D3, E, F, and G) 6. These SNRP proteins form a specific structure to encompass the RNA and to share a conserved SNRP domain which undergoes responsible for the assembling of snRNA in the order sequence 7. The process of splicing on the pre-mRNA plays a pivotal role in the expression of many genes 8, 9. Mutations in splicing factors or alterations in the expression of splicing mechanical elements will affect the splicing patterns, which may lead to the occurrence of many tumors 10-12. Small Nuclear Ribonucleoprotein Polypeptides B and E (SNRPB, SNRPE), both the core components of the spliceosome, were significantly overexpressed in HCC tissues and associated with worse prognosis 13, 14. Additionally, SNRPB has been reported as a prognostic marker for non-small cell lung cancer, cervical cancer, glioma, etc 15-17. SNRPD1 was a crucial gene that regulates the assembly of the pluripotency-specific spliceosome and acquires and maintains pluripotency, and its high protein expression in somatic cells was related to kidney damage and pulmonary hypertension in patients with systemic lupus erythematosus 18, 19. It has been reported that SNRPD1 was significantly overexpressed in neuroblastoma and was an important therapeutic target through regulating the process of mitosis 20. However, no previous studies have investigated the role of SNRPD1 and its prognostic value in HCC.

In this present study, we analyze the mRNA and protein expression of SNRPD1 in the Gene Expression Omnibus (GEO) and the Human Protein Atlas databases, respectively. We explored the gene function by performing gene ontology (GO) and The Kyoto Encyclopedia of Genes and Genomes (KEGG) analysis for co-expressed genes of SNRPD1 21. Next, we investigated the association between the SNRPD1 expression and clinical outcomes in the GSE14520 dataset and 154 HCC cohorts. Then, the luciferase report assay revealed SNRPD1 is the direct target gene of miR-100. Finally, SNRPD1 may be an oncogene to impact the occurrence and development of HCC by regulating the mTOR signaling pathway and autophagy.

## 2. Materials and methods

### 2.1. Gene expression analysis

Gene Expression Profiling Interactive Analysis (GEPIA), a Web server for analysis of the mRNA expression data from The Cancer Genome Atlas (TCGA), was used to analyze the expressed distribution of SNRPD1 in BodyMap and analyze the association of the SNRPD1 expression with tumor stages and survival 22. We used the TNMplot database to comprise SNRPD1 expression in normal, tumor, and metastatic tissues of HCC patients. The Cancer Cell Line Encyclopedia project (CCLE) database was used to investigate the association of SNRPD1 mRNA levels with different liver cancer cell lines. We explore the SNRPD1 protein expression in HCC and adjacent normal tissues from the Human Protein Atlas database 23. We queried the genetic alteration information of the SNRPD1 in cBioPortal for Cancer Genomics and analyzed the correlations between SNRPD1 alteration with prognosis 24.

### 2.2. Prognostic analysis using SNRPD1 expression and clinicopathological data in HCC patients

We downloaded the GSE14520 dataset from the GEO database to study the relationship between the expression of SNRPD1 and the clinical outcome of HCC patients 25. Overall survival (OS) was defined as the time interval between surgery and death or between surgery and the last observation point. Recurrence-free survival (RFS) was defined as the time interval between the date of surgery and the date of diagnosis of any type of recurrence 26. To investigate the prognostic role of SNRPD1, we performed IHC using 154 HCC specimens collected from HCC patients who underwent hepatectomy from January 2012 to December 2013 in 900 Hospital of the Joint Logistics Team. We procured the last follow-up on December 31, 2018. Child-Pugh classification and the 2010 International Union Against Cancer Tumor-Node-Metastasis (TNM) classification system was used to evaluate the liver function and tumor stages, respectively 27, 28. The including criteria of patients are the following: only one tumor node and no metastasis, Child-Pugh class A, no cancer radiotherapy or chemotherapy history before the operation, postoperative pathology confirmed as HCC. Patients younger than 18 years old, who died from non-tumor causes within 1 week after surgery and underwent repeat hepatectomy were excluded from our study. We obtained clinicopathological data of patients from the medical record system of the hospital. Survival information was acquired from the follow-up record and the Social Security Death Index. This study was performed according to the relevant medical ethics regulations and approved by the Human Research Ethics Committee of 900 Hospital of the Joint Logistics Team (Fuzhou, China). All participants gave written informed consent prior to surgery and collection of the specimens.

### 2.3. Analysis of Immunohistochemical staining

154 paraffin-embedded with HCC tissue samples were cut into 4 μm sections, and then fixed them on microscope slides. The tissue sections were deparaffinized with different concentrations of malondialdehyde and rehydrated with ethanol. Antigen retrieval was performed by boiling the slices in sodium citrate buffer (pH 6.0) for 30 minutes. The sections were incubated in 3% H_2_O_2_ for 10 minutes to inhibit endogenous peroxidase and then washed 3 times in phosphate-buffered saline (PBS). Next, polyclonal rabbit anti-SNRPD1 antibody (1:100; ab233115, UK) was added dropwise to the slices, incubated at 4°C overnight, and washed 3 times in PBS. Then, the sections were incubated with secondary antibodies (1:50,000; KIT-5010; anti-rabbit/mouse IgG; China Fuzhou Maixin Biotechnology Development Co., Ltd.) for 30 minutes at room temperature. Next, the sections were stained with 3,3'-diaminobenzidine and substrate chromogen (Dako) for 2 minutes at room temperature and counterstained with hematoxylin for 40 seconds. The negative control group used PBS instead of the anti-SNRPD1 antibody. CX41 microscope (Olympus, Japan) was used to view the IHC staining. The positive IHC staining was independently evaluated by two independent pathologists who did not have any patient information in advance. According to the human protein atlas database, the protein expression of SNRPD1 was mainly detected in the nucleus. We used a semi-quantitative scoring system to evaluate the protein expression of SNRPD1. When no positive cells were detected, scored 0; <=10% positive cells, scored 1; 11%-25% positive cells, scored 2; and when 26%-50% positive cells were detected, scored 3, 51%-75% positive cells, scored 4, more than 75% positive cells, scored 5. A score of 0, 1, and 2 indicates low SNRPD1 expression, whereas a higher score indicates high expression.

### 2.4. GO and KEGG analysis and PPI network construction

We analyzed the co-expressed genes of SNRPD1 in the cBioportal database and the LinkedOmics (http://www.linkedomics.org/login.php) 29, respectively. Then, the overlapping co-expressed genes analyzed by two databases with Pearson's value greater than 0.6 were identified as SNRPD1 co-expressed genes. Next, we explored the gene function of SNRPD1 by performed GO and KEGG enrichment analysis on overlapping SNRPD1 co-expressed genes using the Functional Annotation Tool in the Database for Annotation, Visualization and Integrated Discovery (DAVID, https://david.ncifcrf.gov/) database 30. The protein-protein interaction (PPI) network was constructed in the STRING database (https://www.string-db.org/) with the minimum required interaction score was set as 0.9 (highest confidence) 31. Finally, the PPI network was visualized in Cytoscape software.

### 2.5. GSEA

The normalized gene expression RNAseq data was downloaded from the UCSC Xena database 32. The 374 HCC specimens were divided into high expression groups and low expression groups with the median of SNRPD1 expression as the critical point. Then, the enrichment analysis was performed on these expressed data using GSEA software (V4.1.0) 33. In this process, the KEGG gene sets (c2.cp.kegg.v7.0.symbols.gmt) was selected as the functional gene set, other parameters as the default settings. The pathway of gene enrichment with a normal p-value<0.05 and FDR q-value<0.25 has the significance of the statistics.

### 2.6. Analysis of miRNAs related to SNRPD1 expression in HCC

We queried the miRNAs associated with SNRPD1 expression in the "cBioPortal", "LinkedOmics", "MIRWork", and "TargetScan" databases, respectively, then used Venny software to screen out the overlapping miRNAs. Next, we analyzed the correlations between overlapping miRNAs expression and the clinicopathological outcomes in the LinkedOmics database. We also performed the Kaplan-Meier curve and log-rank test analyses in the Kaplan-Meier plotters database to evaluate the relationship between miRNAs and OS of patients with HCC.

### 2.7. Cell culture and plasmid transfection

We purchased the human normal hepatocyte cell line LO2 from the Chinese Academy of Sciences Committee Type Culture Collection cell bank (Shanghai, China). The HCC cell lines Huh7 and HepG2 were purchased from the American Type Culture Collection (ATCC, USA). All these cell lines were cultured in DMEM basal medium (Hyclone, SH30022.01) supplemented with 100 U/mL penicillin-100 μg/mL streptomycin (Hyclone, SV30010) and 10% fetal bovine serum (FBS, Gibco, 10099141). The DMEM basal medium was placed in an incubator at 37 ℃ with 5% CO2 and saturated humidity. Vectors used in cell transfection including siRNA of SNRPD1, siRNA negative control (si-NC), miR-100 mimic, and mimic negative control (mimic-NC) and were synthesized by Zolgene (Fuzhou, China).

### 2.8. Quantitative polymerase chain reaction (qRT-PCR)

We used RNAiso Plus (TaKaRa, 9109, China) to extract the total RNA of the cells and then used gDNA Purge (Novoprotein, E047-01A, China) to synthesize cDNA based on the manufacturer's instruction. Then, the qPCR was performed to calculate the mRNA expression of SNRPD1 and miR-100 with NovoStart® SYBR qPCR SuperMix Plus (Novoprotein, E096-01B, China) and 7300 Real-Time PCR System (Applied Biosystems, USA). GAPDH and U6 were used as internal controls for SNRPD1 and miR-100, respectively. The primers for GAPDH, U6, SNRPD1, and miR-100 were synthesized by SunYa (Fuzhou, China). The relative expression of SNRPD1 and miR-100 mRNA was determined by using the Ct value and the 2^-ΔΔCt^ method.

### 2.9. Luciferase reporter assay

We used luciferase reporter assay to validate the interaction between SNRPD1 and miR-100. The wild-type (WT) and mutant (MUT) SNRPD1 3'-UTR were designed and inserted into psicheck2.0 vectors (Zolgene, ZVE2012, China). Subsequently, HepG2 cells were co-transfected by combined psicheck2.0 vectors with miR-100 mimic or mimic NC using Lipofectamine 3000 (Invitrogen, Carlsbad, CA, USA). The HCC cells transfected by empty vectors defined as the mock group. Then, the dual-luciferase assays were carried out after incubation for 48h to detect the luciferase activity in transfected cells using Duo-LiteTM Luciferase Assay System (Vazyme Biotech Co.,Ltd, China) according to the manufacturer's recommendations.

### 2.10. Western blot analysis

We lysed the HepG2 cells transfected with si-SNRPD1 or si-NC using RIPA buffer (Meilunbio, China) and incubated on ice for 30mins then extracted the total protein. We separated the total protein by 10% SDS-PAGE (Beyotime, China), then transferred the separated protein to PVDF membrane (Millipore, USA), and then blocked it in 5% non-fat skimmed milk powder (BBI, China) at room temperature for 2 hours. Next, we incubated the membranes with the primary antibodies (SNRPD1: Abcam, ab233115, UK; mTOR: Proteintech, 66888-1-Ig, China; LC3: Abcam, ab192890, UK) overnight at 4°C. Thereafter, we incubated the membranes with the second antibodies (HRP-conjugated Affinipure Goat Anti-Mouse/Rabbit IgG: Proteintech, SA00001-1/SA00001-2, China) for 1 h at 25 °C. Finally, we used the chemiluminescence imaging system (BIO-RAD, USA) to detect the protein blots, and Image Lab software (BIO-RAD, USA) was used to measure the protein expression value.

### 2.11. Autophagy analysis by transmission electron microscopy (TEM)

After transfecting HepG2 cells with siRNA or si-NC for 48 hours, the cells were fixed in 5% glutaraldehyde and 0.1 mol/L phosphate buffer (pH 7.4) for 2 hours at room temperature. The samples were washed three times with PBS buffer and then fixed with 1% osmium tetroxide and 0.1 mol/L phosphate buffer (pH 7.4) for 1.5 h. Next, the cells were rinsed with distilled water, dehydrated in different concentrations of ethanol, and embedded in epoxy resin. The embedded samples were cut into ultrathin sections with a thickness of 70 nm and then stained with uranyl acetate and lead citrate for 5 mins. We used transmission electron microscopy (HT7700, HITACHI, Tokyo, Japan) to observe ultrastructures of cells undergoing autophagy and obtain the images.

### 2.12. Statistical analysis

Pearson's chi-square test was utilized to compare the categorical variables. The student's t-test was used to compare the normally distributed continuous variables. OS and RFS were analyzed using Kaplan-Meier plots with the log-rank test. ROC curve with AUC was utilized to evaluate the diagnostic significance of SNRPD1 and miR-100 for HCC. All assays were repeated at least 3 times. The statistical analysis was carried out using Stata Statistical Software: SPSS 19 (SPSS Inc., Chicago, IL, USA) and GraphPad Prism 5.0 (GraphPad Software, Inc., San Diego, CA, USA). P<0.05 represented a statistically significant difference unless otherwise stated.

## 3. Results

### 3.1. The high SNRPD1 expression is associated with poor survival in HCC patients in the TCGA database

**Figure 1:**
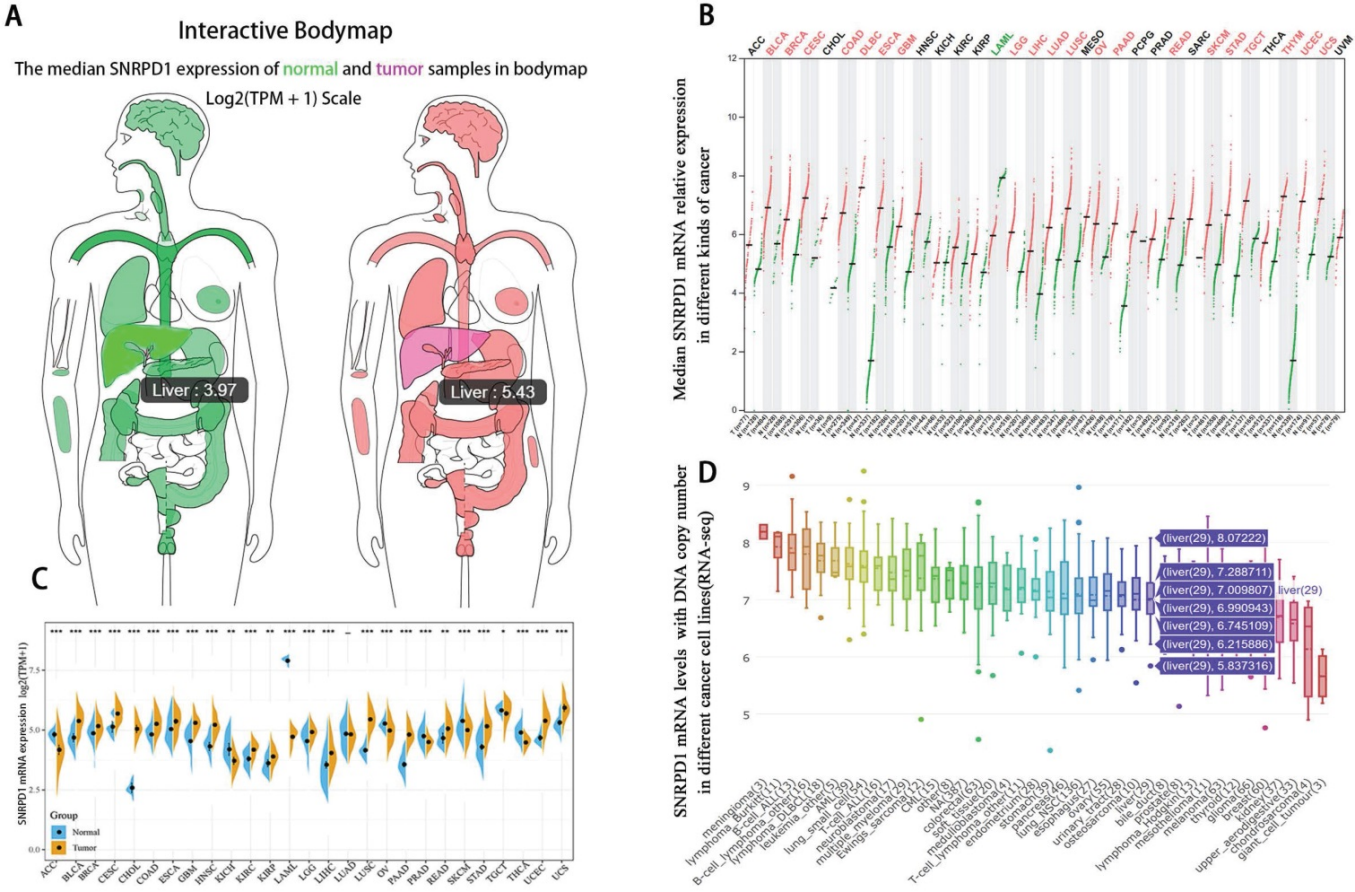
The mRNA expression level of SNRPD1 in various kinds of tumor samples and paired normal tissues. (A) The median SNRPD1 expression of HCC (red, 5.43) and normal liver samples (green, 3.97) in bodymap in the GEPIA. (B) The SNRPD1 expression profile across various kinds of tumor samples (red) and paired normal samples (green) in the GEPIA. Each dots represent the expressed value of each sample. (C)The SNRPD1 expression profile across all tumor samples and paired normal samples in TNMplot database. (D) The SNRPD1 mRNA levels with DNA copy number expressed in different kinds of cancer cell lines in the Cancer Cell Line Encyclopedia (CCLE) database. 29 HCC cell lines have variations in SNRPD1 expression.

**Figure 2:**
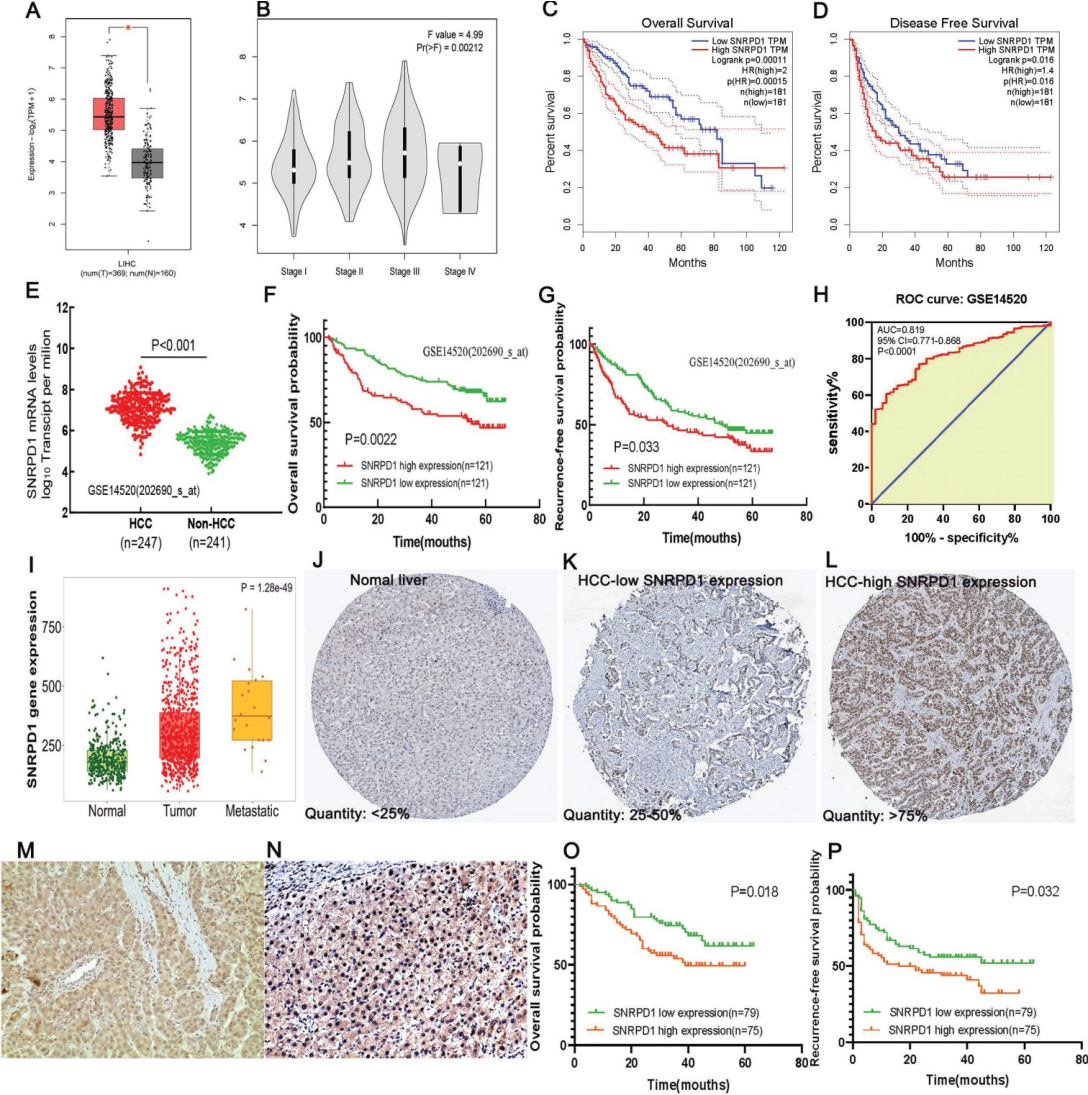
High mRNA and protein expression of SNRPD1 correlates with poor prognosis in HCC patients. (A) The SNRPD1 expression level in HCC samples was significantly higher than normal liver tissues in the GEPIA database(P<0.05). (B) The SNRPD1 expression in HCC samples was incrementally upregulated with increasing tumor stages in the GEPIA database. (C-D) High SNRPD1 mRNA expression correlates with poor overall survival (C) and diseases free survival (D) of HCC patients in the GEPIA database. (E) SNRPD1 expression in HCC tissues was significantly higher than adjacent normal liver tissues in the GSE14520 dataset. (F-G) High SNRPD1 mRNA expression correlates with poor overall survival (F) and recurrence-free survival (G) of HCC patients in the GSE14520 dataset. (H) The receiver operating characteristic (ROC) curve revealed that SNRPD1 expression has a significant diagnosis value on HCC (AUC=0.819, P<0.001). (I) The SNRPD1 mRNA expression was incrementally upregulated in the normal, tumor, and metastatic tissues of HCC patients in the TNMplot database (https://www.tnmplot.com/). (J-L) Representative images of immunohistochemical (IHC) staining of SNRPD1 protein expression in normal liver tissues (J, expression quantity <25%), low expression HCC tissues (K, expression quantity 25-50%), and high expression HCC tissues in the Human Protein Atlas database (L, expression quantity >75%). (M-N) Representative image of IHC staining of SNRPD1 low (M)/high (N) protein expression in tumor tissue from 154 patients with HCC (x200 magnification). (O-P) High SNRPD1 protein expression correlates with poor overall survival (O) and diseases free survival (P) of 154 HCC patients.

The median mRNA expression of SNRPD1 distributed in the tumor tissues in the interactive bodymap is higher than normal tissues (Fig. 1A). Besides, we analyzed the expression of SNRPD1 in a variety of human tumors and paired normal tissues in the TCGA and TNMplot database (Fig. 1B-C). The analysis of CCLE exhibited that 29 hepatocellular carcinoma lines have the copy number variation of SNRPD1 mRNA expression (Fig. 1D). We analyzed the expression of SNRPD1 in HCC tissues and normal tissues in the GEPIA database. The results showed that the expression of SNRPD1 in HCC tissues was significantly higher than normal liver tissues (Fig. 2A), and incrementally upregulated with increasing tumor stages (Fig. 2B). We performed the Kaplan-Meier analysis in the GEPIA database and results demonstrated that high SNRPD1 expression in patients with HCC correlated with shorter overall survival (HR=2, P<0.001; Fig. 2C) and disease-free survival (HR=1.4, P=0.016; Fig. 2D).

### 3.2. The relationship between the SNRPD1 expression and clinical outcomes in patients with HCC in the GEO database

We downloaded the GSE14520 dataset from the GEO database to analyze the relationship between SNRPD1 expression and the clinical outcomes of HCC patients. The mRNA expression of SNRPD1 in the HCC tissues (n=247) was significantly higher than the non-HCC tissues (n=241, P<0.001) (Fig. 2E). The high SNRPD1 mRNA expression was significantly positively correlated to the TNM stage (P=0.025), serum AFP level (P< 0.001), CLIP staging (P<0.001). Gender, age, tumor size, ALT, BCLC staging, multinodular, and cirrhosis were not correlated to the SNRPD1 expression (Table 1). We found that Tumor size (P=0.001), TNM staging (P<0.001), Serum AFP level (P=0.011), BCLC staging (P<0.001), CLIP staging (P=0.001), Multinodular (P=0.023), Cirrhosis (P=0.023), and high SNRPD1 expression (P=0.003) were risk factors for overall survival of HCC by performing Univariate Cox Regression analysis. The Multivariate Cox Regression analysis confirmed that BCLC staging (HR (95%CI): 5.381(2.985-9.700); P<0.001), Multinodular (HR (95%CI): 2.012(1.082-3.740); P=0.027), Cirrhosis (HR (95%CI): 0.206(0.050-0.839); P=0.028) and high SNRPD1 expression (HR (95%CI): 1.968(1.266-3.060); P=0.003) were independent risk factors for overall survival. For RFS, the Gender (P=0.009), TNM staging (P<0.001), BCLC staging (P<0.001), CLIP staging (P=0.016), and high SNRPD1 expression (P=0.034) were risk factors. Meanwhile, Gender (HR (95%CI): 0.516(0.269-0.990); P=0.047), BCLC staging (HR (95%CI): 2.496(1.697-3.672); P<0.001) and high SNRPD1 expression (HR (95%CI): 1.299(0.878-1.920); P=0.042) were independent risk factors of RFS analyzed by Multivariate Cox Regression (Table 2). The Kaplan-Meier analysis and log-rank test analysis demonstrated that high SNRPD1 mRNA expression led to a poor OS (p=0.0022, Fig. 2F) and RFS (P=0.033, Fig 2G) in patients with HCC. The receiver operating characteristic (ROC) curve revealed that SNRPD1 expression has a significant diagnosis value on HCC (AUC=0.819, P<0.001, Fig. 2H). In addition, SNRPD1 mRNA expression incrementally upregulated with normal, tumor, and metastatic tissues analyzed in the TNMplot database (p=1.28e-49, Fig. 2I). Furthermore, the analysis of SNRPD1 protein expression in the Human Protein Atlas database showed that its protein level in HCC samples (Fig. 2K-L) was higher than normal liver tissues (Fig. 2J).

### 3.3. The relationship between the expression of SNRPD1 and clinicopathologic characteristics in 154 HCC patients

As shown in the Figures, our IHC validated that SNRPD1 mainly expressed on the nucleus of HCC cells (Fig. 2M-N). Based on the semi-quantitative scoring system, we divided 154 cases of HCC into high expression groups (n=75) and low expression groups (n=79). The SNRPD1 protein expression level was significantly correlated to Age (P=0.049), TNM staging (P=0.010), Serum AFP level (P=0.001), Tumor differentiation (P=0.002), Vascular invasion (P=0.006), Recurrence (P=0.036), and Survival (P=0.025) (Table 3). Univariate Cox Regression analysis showed that TNM staging (P=0.014), Vascular invasion (P=0.033), and high SNRPD1 protein expression (P=0.015) were risk factors for OS of HCC. The Multivariate Cox Regression analysis confirmed that TNM staging (HR (95%CI): 1.997(1.136-3.511); P=0.016) and high SNRPD1 expression (HR (95%CI): 1.890(1.098-3.255); P=0.022) were independent risk factors for OS. For RFS of HCC, TNM staging (P=0.027), Tumor differentiation (P=0.046), Vascular invasion (P=0.002), Tumor encapsulation (P<0.001), and high SNRPD1 expression (P=0.030) were risk factors. Meanwhile, the multivariate cox regression revealed that TNM staging (HR (95%CI): 1.682(1.049-2.699); P=0.031), Vascular invasion (HR (95%CI): 1.861(1.122-3.087); P=0.016), Tumor encapsulation (HR (95%CI): 0.209(0.129-0.338); P<0.001), and high SNRPD1 protein expression (HR (95%CI): 1.735(1.070-2.813); P=0.026) were independent risk factors (Table 4). Finally, The Kaplan-Meier analysis showed that high SNRPD1 protein expression led to a poor OS (p=0.018, Fig. 2O) and RFS (P=0.032, Fig 2P) in patients with HCC.

Abbreviations: AFP - alpha fetoprotein, TNM - tumor, node, metastasis. *P-Value<0.05 were considered statistically significant.

### 3.4. SNRPD1 alterations analysis in TCGA database and gene function analysis through GO, KEGG, and GSEA

**Figure 3:**
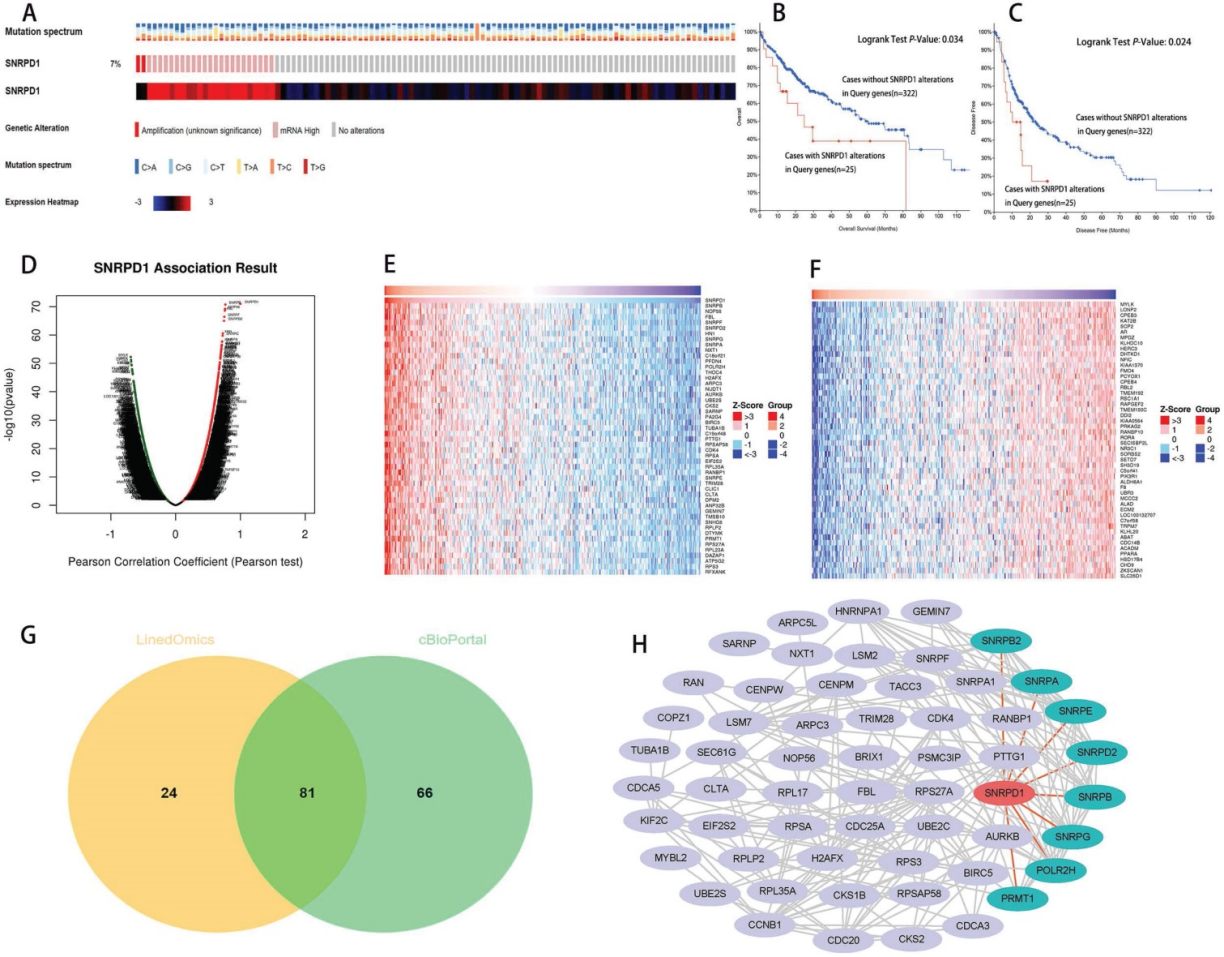
Alteration analyzed of SNRPD1 and identification of co-expressed genes and construction of protein-protein interaction (PPI) network. (A) The mutation spectrum, alteration and expression heatmap of SNRPD1 in a cohort of 349 HCC patients in the cBioPortal database. 7% cases of this cohort exhibited alteration, including 2 cases of amplification, and 23 cases of mRNA high expression. (B-C) The overall survival (B), and the disease-free survival (C) of HCC patients with SNRPD1 alterations (n=25), was poorer than without SNRPD1 alterations (n=324). (D-F) SNRPD1 expression associated target genes analysis in the LinkedOmics database. (D) Volcano chart exhibited SNRPD1 expression positively/negatively correlated significant genes. (E) The top 50 genes that are positively associated with SNRPD1 expression. (F) The top 50 genes that are negatively associated with SNRPD1 expression. (G)81 overlapping correlated genes with Spearman's Correlation greater than 0.6 were screened as SNRPD1 co-expressed genes. (H) PPI network for 81 co-expressed genes of SNRPD1 was constructed and visualized. SNRPA, SNRPB, SNRPB2, SNRPD2, SNRPE, SNRPG, POLR2H, and PRMT1 protein can interact with SNRPD1.

We queried the SNRPD1 alterations in a cohort of 347 HCC patients (TCGA, PanCancer Atlas) in the cBioportal database. The result showed that SNRPD1 altered in 25 (7%) of queried HCC patients, including 2 cases of amplification, and 23 cases of mRNA high expression (Fig. 3A). In addition, the Kaplan-Meier curve shows that the OS (P=0.034, Fig. 3B), as well as the disease-free survival (P=0.024, Fig. 3C) of HCC patients with SNRPD1 alterations (n=25), was poorer than without SNRPD1 alterations (n=322).

**Figure 4:**
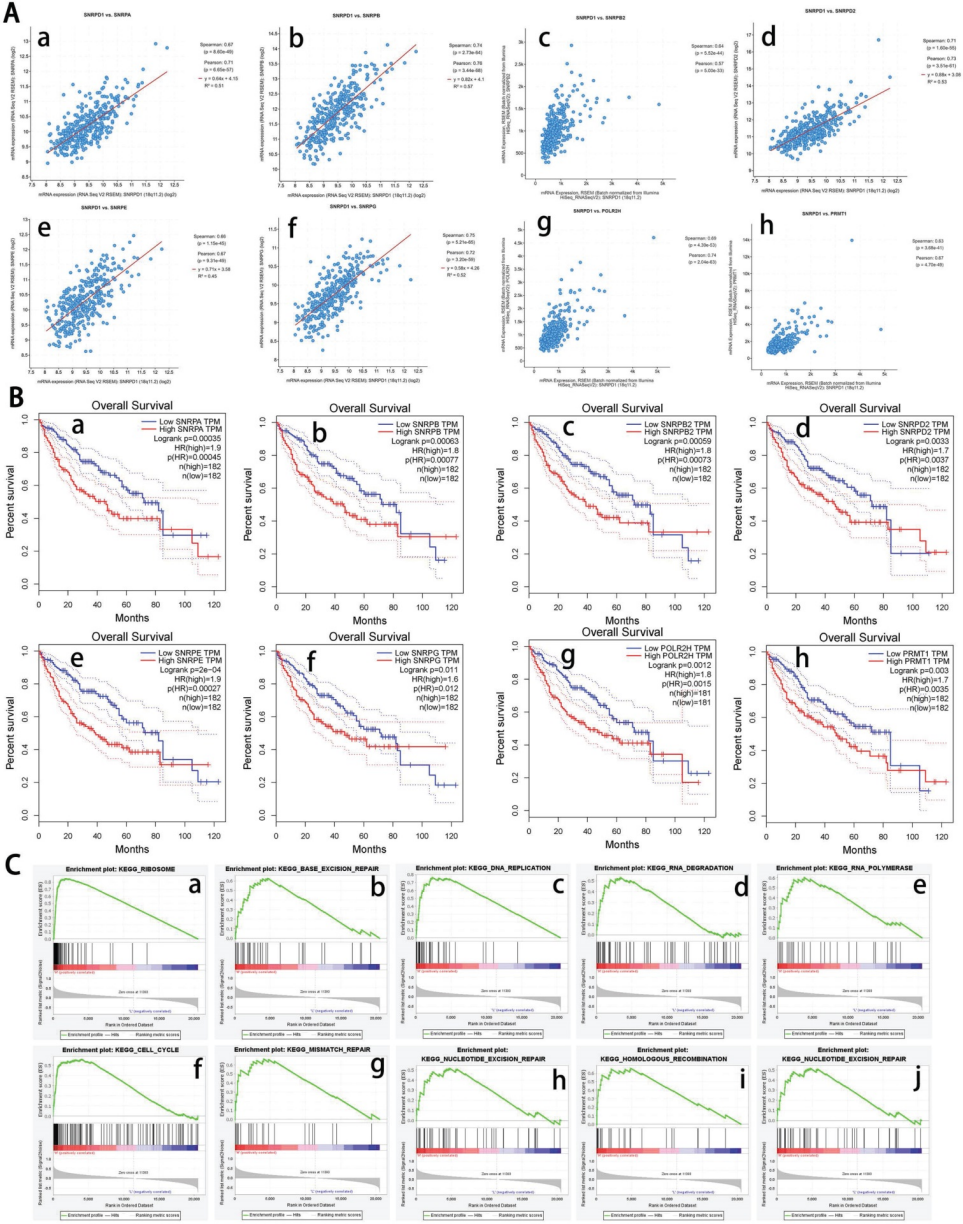
Survival analysis of co-expressed genes interacted with SNRPD1and KEGG pathways analysis using GSEA. (A) Correlation between SNRPD1 and SNRPA (a, r=0.71), SNRPB (b, r=0.76), SNRPB2 (c, r=0.64), SNRPD2 (d, r=0.73), SNRPE (e, r=0.67), SNRPG (f, r=0.75), POLR2H (g, r=0.74), and PRMT1 (h, r=0.67), all P<0.05. (B) The survival analysis showed that the mRNA expression of SNRPA (a), SNRPB (b), SNRPB2 (c), SNRPD2 (d), SNRPE (e), SNRPG (f), POLR2H (g), and PRMT1 (h) were significantly related to the OS in HCC patients (all P<0.05). (C) The main enriched KEGG pathways of SNRPD1 using GSEA. RIBOSOME (a), BASE EXCISION REPAIR (b), DNA_REPLICATION (c), DNA REPLICATION (d), RNA POLYMERASE (e), CELL CYCLE (f), MISMATCH REPAIR (g), NUCLEOTIDE EXCISION REPAIR (h), HOMOLOGOUS RECOMBINATION (i), BLADDER CANCER (j).

We analyzed the co-expressed genes of SNRPD1 using the HCC dataset in the cBioportal database and the LinkedOmics, respectively. 11459 positively and 8627 negatively genes correlated with SNRPD1 protein expression in a cohort of 371 HCC patients from the LinkedOmics database expression were investigated (Fig. 3D). The top 50 positively and negatively correlated genes were exhibited using the heat map, respectively (Fig. 3E-F). 81 overlapping correlated genes with Spearman's Correlation greater than 0.6 obtained in the LinkedOmics and cBioportal database were screened as SNRPD1 co-expressed genes (Fig. 3G). Next, we explored the gene function of SNRPD1 by performing GO and KEGG analysis on 81 SNRPD1 co-expressed genes in the DAVID database. The GO analysis revealed that the SNRPD1 co-expressed genes mainly enriched in Cell division, Nuclear import, mRNA splicing via spliceosome, Mitotic nuclear division, Spliceosomal snRNP assembly, and Regulation of cell cycle, etc. The KEGG analysis revealed that SNRPD1 co-expressed genes were mainly enriched in the signal pathway of Spliceosome, Ribosome, and Cell cycle (Table 5). Then, we explored the significant interactions of SNRPD1 with 81 co-expressed genes using the STRING database, with a confidence score of >0.900 (highest confidence). Ultimately, a PPI network with 57 nodes and 212 edges was constructed and visualized in the Cytoscape software. The PPI network showed that the SNRPA, SNRPB, SNRPB2, SNRPD2, SNRPE, SNRPG, POLR2H, and PRMT1 protein can interact with SNRPD1 (Fig. 3H). The Spearman rank correlation test in the cBioPortal database confirmed that SNRPA, SNRPB, SNRPB2, SNRPD2, SNRPE, SNRPG, POLR2H, and PRMT1 protein positively correlated with SNRPD1 (all Spearman>0.63, P<0.05) (Fig. 4A). Furthermore, the survival analysis performed in the GEPIA database showed that SNRPA, SNRPB, SNRPB2, SNRPD2, SNRPE, SNRPG, POLR2H, and PRMT1 mRNA expression associated with OS of HCC patients (Fig. 4B).

To further explore gene functions and potential pathways regulating the occurrence and development of HCC, we performed GSEA enrichment analysis using gene expression data downloaded from the TCGA database. The results showed that the KEGG signaling pathways related to occurrence and development of HCC including "ribosome", "base excision repair", "DNA replication", "cell cycle", and "mismatch repair", etc. (Fig. 4C). The GSEA result also showed that the genes enriched in these KEGG pathways were significantly altered in the SNRPD1 aberrantly high expression group (Table 6). In summary, we have the reasonable consideration that SNRPD1 may regulate the tumorigenesis and development of HCC through these signaling pathways.

### 3.5. miR-100 as a potential HCC suppressor via negatively targeting SNRPD1 expression

**Figure 5:**
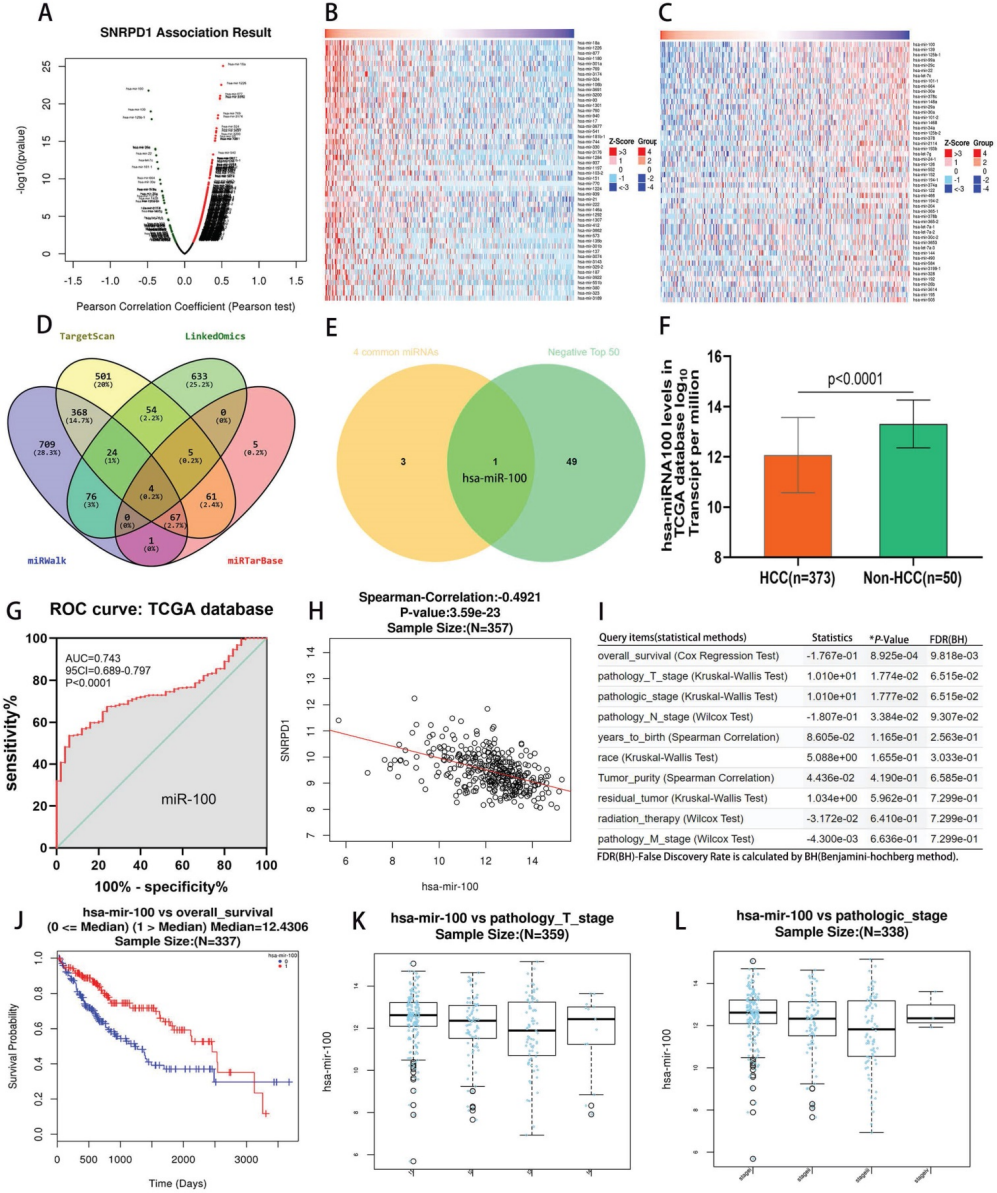
Analysis of association between miRNAs and SNRPD1 expression in the TCGA database. (A-C) SNRPD1 expression associated miRNAs analysis in the LinkedOmics database. (A) Volcano chart exhibited SNRPD1 expression positively/negatively correlated significant miRNAs. (B) The top 50 miRNAs that are positively associated with SNRPD1 expression. (C) The top 50 miRNAs that are negatively associated with SNRPD1 expression. (D) 4 overlapping miRNAs interact with SNRPD1 obtained from the “LinkedOmics”, “miRWalk”, “TargetScan”, and “miRTarBase” databases. (E) The Venny diagram exhibited that hsa-miR-100 overlapping in “4 Common miRNAs” and “SNRPD1 Negatively Correlated Significant miRNAs (top 50)”. (F) The expression of miR-100 in HCC tissues (n=373) was significantly lower than that in non-HCC tissues (n=50) in the TCGA database. (G) The receiver operating characteristic (ROC) curve revealed that miR-100 expression has a significant diagnosis value on HCC (AUC=0.743, P<0.0001). (H) Scatter plot visualizing that hsa-mir-100 negatively significantly correlated with SNRPD1 expression in the HCC patients (Spearman-correlation: -0.4921, P=3.59e-23). (I) Association of miR-100 expression with clinicopathologic outcomes in HCC patients in the LinkedOmics database. (J) The survival analysis showed that low hsa-miR-100 mRNA expression were significantly associated with the poor overall survival in HCC patients (P=8.925e-04). (K) Hsa-miR-100 expression was correlated with pathology_T_stage (P = 1.774E-02). (L) Hsa-miR-100 expression was correlated with pathologic_stage (P = 1.777E-02).

We investigated the 614 positively and 181 negatively correlated microRNAs related to SNRPD1 expression in the LinkedOmics database (Fig. 5A). The top 50 positively and negatively correlated microRNAs were exhibited in the heat map, respectively (Fig. 5B-C). Next, we searched for microRNAs related to SNRPD1 expression in the miRWalk, TargetScan, and miRTarBase databases, respectively. Then, we obtained four overlapping microRNAs from the above four databases, namely miR-100, miR-665, miR-940, miR-3911 (Fig. 5D). We then used the Venn Diagrams to intersect the four overlapping microRNAs with the top 50 negatively correlated microRNAs, the remaining one microRNA (miR-100) (Fig. 5E). We studied the expression of miR-100 in the TCGA database and found that the expression of mir-100 in HCC was significantly lower than that in non-HCC tissues (P<0.0001, Fig. 5F). The ROC curve revealed that miR-100 expression has a significant diagnosis value on HCC (AUC=0.743, P<0.0001, Fig. 5G). There was a significantly negative correlation between the expression of mir-100 and SNRPD1 in HCC patients (r=-0.4921, P=3.59e-23) (Fig. 5H). We also analyzed the relations between the expression of miR-100 and the clinical outcome of HCC patients in the Linkedomics database (Fig. 5I). The results showed that low miR-100 expression was associated with the overall _ survival (P=8.925e-04, Fig. 5J), pathology_ T_ stage (P=1.774e-02, Fig. 5K), pathologic_ stage (P=1.777e-02, Fig. 5L), and pathology_ N_ Stage (P=3.384e-02), etc.

### 3.6. SNRPD1 is the direct target gene of miR-100 in HCC cells

**Figure 6:**
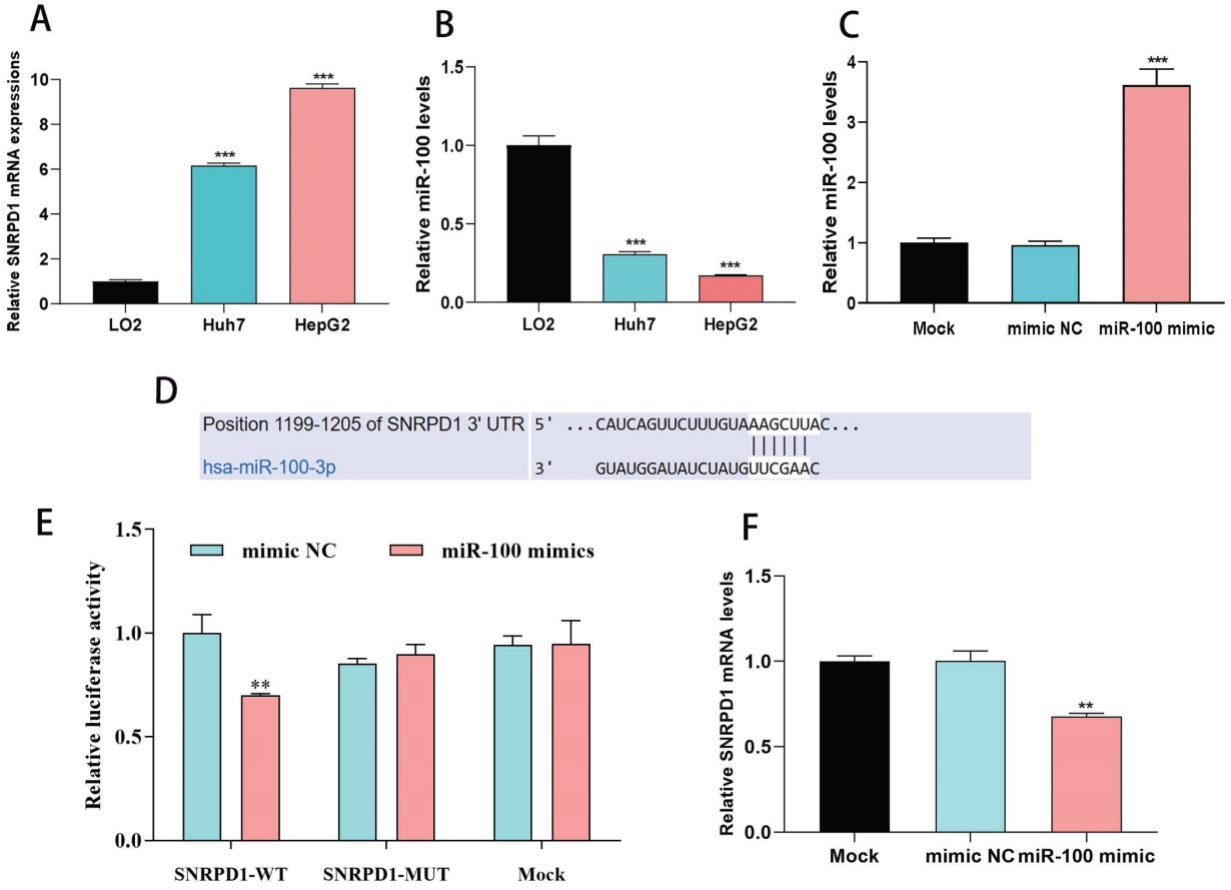
Correlations between miR-100 and SNRPD1 expression in HCC cells. (A) mRNA expression of SNRPD1 in HCC cells was significantly higher than normal liver cells (P<0.001). (B) mRNA expression of miR-100 in HCC cells was significantly lower than normal liver cells (P<0.001). (C) Expression of miR-100 was significantly upregulated by cell transfection with miR-100 mimic (P<0.001). (D) Predicted complementary sequences of hsa-miR-100-3p in 3'-UTR of SNRPD1. (E) The HepG2 cells transfected by miR-100 mimic were significantly decreased luciferase activity of WT 3'-UTR of SNRPD1 (P<0.01). (F) The upregulation of miR-100 expression in HepG2 cells by transfected miR-100 mimics resulted in significantly decreased expression of SNRPD1 (P<0.01).

We used one normal liver cell line (LO2) and two HCC cells line (Huh7, HepG2) to investigate the effect of miR-100 on the expression of SNRPD1. The results showed that SNRPD1 (Fig. 6A) expression was significantly upregulated while the expression of miR-100(Fig. 6B) was significantly downregulated in Huh7 and HepG2 cells line when compared with the LO2 cells line (all P<0.001). We verified the relations between miR-100 and SNRPD1 expression using qRT-PCR. The results exhibited a significantly upregulated miR-100 expression (Fig. 6C) and a significantly downregulated SNRPD1 expression (Fig. 6D) after the miR-100 mimic was transfected into HepG2 (all P<0.001). We further validated the associations between miR-100 with SNRPD1 expression using luciferase reporter assay and found that the HepG2 cells transfected by miR-100 mimic were decreased luciferase activity of WT 3'-UTR of SNRPD1, but mutant SNRPD1-expressing cells and mock group cells showed no luciferase activity decrease (Fig. 6E-F). Overall, these results indicated that SNRPD1 was the direct target gene of miR-100 in HCC and was negatively regulated by miR-100.

### 3.7. SNRPD1 knockdown leads to the mTOR signaling pathway downregulated in HCC cells

**Figure 7:**
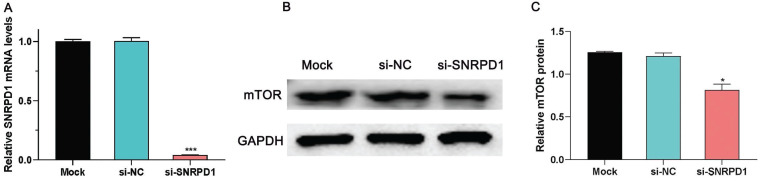
Effect of SNRPD1 on mTOR signaling pathway. (A) The siRNA of SNRPD1 (si-SNRPD1) successfully inhibited mRNA expression of SNRPD1 in HepG2 cells. (B) The western blot analysis exhibited the mTOR protein expression in HepG2 cells. (C) si-SNRPD1 significantly decreased the mTOR protein expression in HepG2 cells.

Previous studies have found that knockdown the expression of SNRPE, another core SNRP spliceosomal protein, can inhibit the mTOR pathway in breast cancer SKBr-3 cell lines 3. Therefore, we hypothesized whether SNRPD1 regulates the occurrence and development of HCC through the mTOR pathway. In this present study, we briefly investigated the effect of SNRPD1 mRNA expression on the activity of the mTOR signaling pathway in the HepG2 cell line. As shown in Figure. 7A, the SNRPD1 expression level was successfully inhibited by the siRNA of SNRPD1 (P<0.001, Fig. 7A). The western blot analysis showed that the mTOR protein level of si-SNRPD1 groups was significantly decreased compared with the Mock and si-NC groups (P<0.05, Fig. 7B-C). All these results indicated that mTOR signaling pathway was blocked in HepG2 cells due to low expression of SNRPD1.

### 3.8. SNRPD1 knockdown promotes autophagy of HCC cells

**Figure 8:**
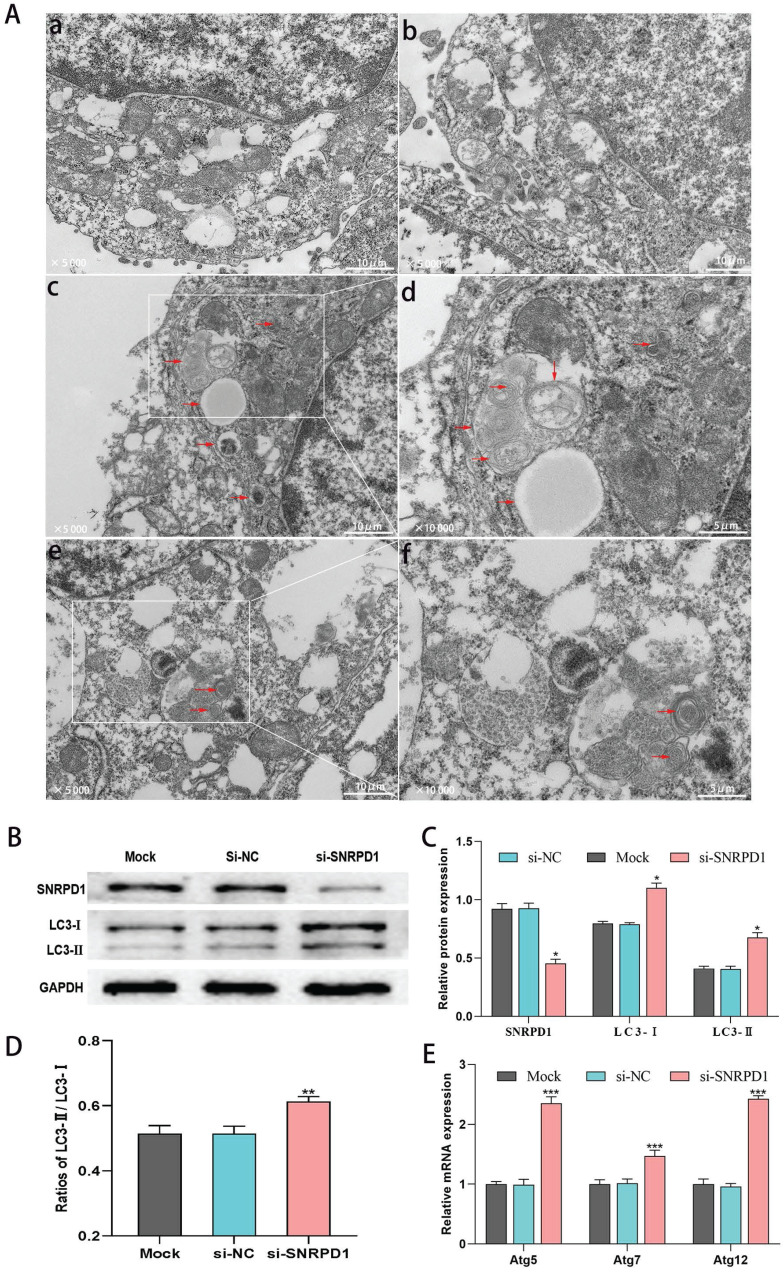
siRNA of SNRPD1 (si-SNRPD1) promotes autophagy in HepG2 cells. (A) Transmission electron microscopy (TEM) was used to detect the autophagic events characterized. HepG2 cells were transfected with si-SNRPD1 or without si-SNRPD1 (si-NC). Compared with si-NC HepG2 cells (a, b), autophagosomes and autolysosomes were mainly observed in si-SNRPD1 cells (c, d, e, f). HepG2 cells transfected by si-SNRPD1 or si-NC were shown at 2 magnifications, x5,000 (a, b, c, e) and x10,000 (d, f). (B) The western blot analysis exhibited the LC3-I and LC3-II protein expression in HepG2 cells transfected by si-SNRPD1 or si-NC. (C-D) LC3-I, LC3-II protein expression (C, P<0.05), and LC3-II/LC3-I ratio (D, P<0.01) significantly increased in si-SNRPD1 transfected cells. (E) The real-time PCR analysis implicated that Atg 5, Atg 7, and Atg 12 mRNA levels significantly elevated in si-SNRPD1 transfected cells (all P<0.001).

We used transmission electron microscopy (TEM) to detect the ultrastructural details of autophagosomes to evaluate the influence of SNRPD1 knockdown on autophagy. As shown in Figure. 8A, HepG2 cells transfected with si-SNRPD1 exhibited a remarkable increase number of autophagosomes and autolysosomes, compared with si-NC cells (Fig. 8A). At the same time, the rates of autophagosomes found in cells transfected with si-SNRPD1 and si-NC were 11% and 4%, respectively. We next analyzed the effect of si-SNRPD1 transfection on the expression of Microtubule-Associated Protein 1 Light Chain 3 (LC3), commonly used as a critical protein involved in the process of autophagy and directly participated in the formation of autophagosomes 34, 35. Consistent with the results of TEM, western blotting analyses showed that si-SNRPD1 transfected in HepG2 cells significantly decreased SNRPD1 protein level, but significantly increased expression of LC3-I, LC3-II protein, and LC3-II/LC3-I ratio (Fig. 8B-D). The results of real-time PCR analysis implicated that Atg 5, Atg 7, and Atg 12 mRNA levels significantly elevated in si-SNRPD1 transfected cells (Fig. 8E). Overall, these data indicated that SNRPD1 knockdown might promote autophagy of HCC cells.

## 4. Discussion

Spliceosome was responsible for participating in the splicing of the pre-messenger RNA, such as removing introns and then the connection of exons in a certain order 3-5. The process of RNA splicing governs many aspects of cellular proliferation, survival, and differentiation. Alteration of this process has been proved implicating in many human cancers 36-38. SNRPB and SNRPE, both the core components of the spliceosome, were significantly overexpressed in HCC tissues and associated with a worse prognosis 13, 14. SNRPD1 also was a crucial gene that regulates the assembly of the pluripotency-specific spliceosome and acquires and maintains pluripotency 19. Ming Yi and colleagues constructed a gene co-expression network using weighted gene co-expression Network Analysis showed that SNRPD1 was a predictive biomarker of lung adenocarcinoma due to its high expression was associated with poor prognosis 39. Studies have shown that SNRPD1 overexpression promotes the development of breast cancer by cooperating with genes involved in the cell cycle, mitosis, and chromatin replication. And silencing SNRPD1 in breast cancer cells may cause tumor cell growth to stop and cell cycle arrest in the G0/G1 stage 40. However, no previous study has addressed the role of SNRPD1 in HCC and its prognostic and diagnostic value. This study is the first systematic investigation of the associations between SNRPD1 expression in mRNA and protein level with clinical outcomes, diagnostic and prognostic value in HCC patients.

SNRPD1 mRNA expression in HCC was significantly higher than in normal liver tissues. Otherwise, its expression is incrementally upregulated with increasing tumor stages. Furthermore, the investigation of protein expression of SNRPD1 in the human protein atlas database showed that its expression higher than normal liver tissues, which was validated by our IHC of 154 HCC patients. Higher mRNA and protein expression of SNRPD1 were both associated with poor OS and RFS of HCC patients. The ROC curve revealed that SNRPD1 mRNA expression has a significant diagnostic value on HCC.

High SNRPD1 mRNA expression was significantly positively correlated to the TNM stage, serum AFP level, CLIP staging. The multivariate regression analysis confirmed that the high SNRPD1 mRNA expression was an independent risk factor for OS and RFS in HCC patients. The analysis of clinicopathologic characteristics in 154 HCC patients revealed that high SNRPD1 protein expression was significantly correlated to TNM staging, serum AFP level, tumor differentiation, vascular invasion, recurrence, and survival. IHC was routine pathological examination after liver cancer resection. Our result demonstrated that high SNRPD1 protein expression was an independent risk factor for OS and RFS of HCC patients. Therefore, postoperative SNRPD1 IHC examination may help to predict the prognosis and recurrence of patients with HCC.

Mutations in genes encoding the components of the splicing machinery were reported in carcinoma from various origins, such as hematologic malignancies, breast, and colon 41, 42. Volker and colleagues showed that mutations in SNRPD1 lead to a significant dominance of T cells targeting the mutant epitope in melanoma patients, leading to the development of anti-tumor immunity 43. We queried the SNRPD1 alterations in a cohort of 347 HCC patients and found that 25 (7%) of queried HCC patients has the alterations. Notably, the OS and disease-free survival of HCC patients with SNRPD1 alterations were significantly lower than those without the alterations. Therefore, exploring the cause of SNRPD1 alterations may promote finding a new method for the treatment of HCC by targeting the expression and alteration of SNRPD1.

To investigate the role of SNRPD1 in the occurrence and development of HCC, GO and KEGG enrichment analysis on co-expressed genes of SNRPD1 was performed. GO analysis showed that the biological processes of SNRPD1 implicated included cell division, mRNA splicing via spliceosome, mitotic nuclear division, etc. We noticed that the KEGG pathways of DAVID and GESA indicated that SNRPD1 was involved in regulating the cell cycle, consistent with the previous study. SNRPD1 promotes the progression of breast cancer by regulating the cell cycle 40. Based on the above research results, it is reasonable to hypothesize that the overexpression of SNRPD1 may lead to the overexpression of genes that promote the cell cycle by causing changes in RNA splicing and promote the progression of HCC, which was worth further study.

MicroRNAs (miRNAs), highly conserved small non-protein-coding RNAs, play a crucial role in negatively regulating various cancers through targeting a wide range of target genes 44, 45. Therefore, the identification of miRNAs that regulating the occurrence and development of HCC may provide new methods for the diagnosis and treatment of HCC. Ke R and colleagues found that miR-22 was significantly down-regulated and promoted the progress of HCC by negatively targeting the expression of HNRNPA1 46. Previous studies have found that miR-100 plays a role in suppressor in HCC 47, 48. Our research-based on bioinformatics analysis found that miR-100 directly targets and negatively regulates the expression of SNRPD1 in HCC. The miR-100 expression was significantly down-regulated in HCC. The ROC curve shows that miR-100 has a significant diagnostic value for HCC. Furthermore, low miR-100 expression was associated with poor overall survival, pathologic stage, pathology T stage, and pathology N stage in HCC. Our luciferase reporter assay revealed that SNRPD1 was the direct target gene of miR-100 in HCC cells. This result was consistent with bioinformatics.

Previous studies found that SNRPD1 knockdown can inhibit the mTOR pathway and promote autophagy in breast cancer SKBr-3 cell lines 3. Previous studies have reported that miR-100 regulate the mTOR signaling pathway in a variety of tumor types. Ye et al. reported that miR-100 downregulates mTOR to suppress the proliferation, migration, and Invasion of prostate cancer Cells 49. Yu et al. demonstrated that miR-100 up-regulation enhanced cell autophagy and apoptosis induced by cisplatin in osteosarcoma by targeting mTOR 50. In addition, Lin et al. reported that miR-100 inhibits cell proliferation in mantle cell lymphoma by targeting mTOR 51. Another study revealed that miR-100 promotes the autophagy of hepatocellular carcinoma cells by inhibiting the expression of the mTOR pathway 52. Therefore, we supposed that SNRPD1 acts as an oncogene in the occurrence and progression of HCC through regulates the mTOR pathway and autophagy. Our western blot analysis showed that mTOR was significantly downregulated after SNRPD1 was knockdown, whereas LC3-I, LC3-II was significantly upregulated, and LC3-II/LC3-I ratio was increased. Furthermore, HCC cells transfected with si-SNRPD1 had a remarkable increase number of autophagosomes and autolysosomes, compared with si-NC cells, which was confirmed by TEM. Overall, all our results indicating that SNRPD1 acts as an oncogene in the occurrence and progression of HCC through regulates the mTOR pathway and autophagy.

## 5. Conclusion

In summary, our study demonstrates that SNRPD1 expression was significantly upregulated in HCC compared with adjacent normal liver tissues. High SNRPD1 expression was associated with poor clinical outcomes. SNRPD1 may regulate the progression of HCC by influencing the process of cell cycle and mRNA splicing via spliceosome, etc. SNRPD1 was the direct target gene of miR-100 in HCC cells. SNRPD1 may as an oncogene regulating the tumorigenesis and progression of HCC through regulates the mTOR pathway and autophagy, suggesting that designing new drugs targeting SNRPD1 may provide new insight and methods for the treatment of HCC.

## Figures and Tables

**Table 1:** Correlation between SNRPD1 expression and clinical outcomes in HCC patients (242 cases, GSE14520 dataset).

Characteristics			SNRPD1 level		
		N	high(n)	low(n)	*P-Value
Gender	Male	211	103	108	0.336
	Female	31	18	13	
Age	>55	75	32	43	0.126
	<=55	167	89	78	
Tumor size	>5cm	88	46	42	0.559
	<=5cm	153	74	79	
TNM stage	I/II	176	83	93	0.025
	III	49	32	17	
Serum AFP level	>300ng/ml	110	73	37	<0.001
	<=300ng/ml	128	47	81	
ALT	>50U/L	100	49	51	0.794
	<=50U/L	142	72	70	
BCLC staging	Yes	225	115	110	0.746
	0-A	173	86	87	
	B-C	52	29	23	
CLIP staging	0	98	36	62	<0.001
	1-5	127	79	48	
Multinodular	Yes	51	29	22	0.270
	No	191	92	99	
Cirrhosis	Yes	19	12	7	0.232
	No	223	109	114	

Abbreviations: AFP - alpha fetoprotein, TNM - tumor, node, metastasis; ALT, alanine aminotransferase. BCLC, Barcelona Clinic Liver Cancer. *P-Value<0.05 were considered statistically significant.

**Table 2:** Univariate and Multivariate Cox Regression analysis of overall survival and Recurrence-free survival in HCC patients (242 cases, GSE14520 dataset).

Variables		Overall survival	*P-Value	Recurrence-free survival	*P-Value
		HR (95%CI)		HR (95%CI)	
Univariate analysis					
Gender	Male vs. female	0.538(0.261-1.110)	0.094	0.424(0.223-0.807)	0.009
Age(years)	>55 vs. <=55	0.759(0.484-1.191)	0.231	1.022(0.714-1.464)	0.905
Tumor size(cm)	>5 vs. <=5	1.953(1.303-2.928)	0.001	0.713(0.504-1.008)	0.055
TNM staging	I/II vs. III	0.275(0.177-0.428)	<0.001	2.215(1.499-3.274)	<0.001
Serum AFP level(ng/ml)	>300 vs <=300	1.686(1.126-2.527)	0.011	0.761(0.543-1.067)	0.113
ALT(U/L)	>50 vs <=50	0.866(0.579-1.295)	0.483	0.724(0.517-1.014)	0.060
BCLC staging	0-A vs. B-C	3.692(2.381-5.726)	<0.001	2.647(1.803-3.888)	<0.001
CLIP staging	0 vs 1-5	2.194(1.384-3.478)	0.001	1.577(1.085-2.234)	0.016
Multinodular	Yes vs. no	0.599(0.385-0.931)	0.023	0.734(0.495-1.088)	0.124
Cirrhosis	Yes vs. no	5.093(1.255-20.671)	0.023	0.499(0.233-1.068)	0.074
SNRPD1	High vs. low	0.535(0.356-0.803)	0.003	0.695(0.496-0.973)	0.034
Multivariate analysis					
Gender	Male vs. female			0.516(0.269-0.990)	0.047
Age(years)	>55 vs. <=55				
Tumor size(cm)	>5 vs. <=5	0.945(0.517-1.727)	0.544		
TNM staging	I/II vs. III	1.362(0.625-2.971)	0.322	0.976(0.524-1.816)	0.780
Serum AFP level(ng/ml)	>300 vs <=300	1.366(0.695-2.686)	0.741		
ALT(U/L)	>50 vs <=50				
BCLC staging	0-A vs. B-C	5.381(2.985-9.700)	<0.001	2.496(1.697-3.672)	<0.001
CLIP staging	0 vs 1-5	1.751(0.787-3.894)	0.274	1.136(0.741-1.742)	0.330
Multinodular	Yes vs. no	2.012(1.082-3.740)	0.027		
Cirrhosis	Yes vs. no	0.206(0.050-0.839)	0.028		
SNRPD1	High vs. low	1.968(1.266-3.060)	0.003	1.299(0.878-1.920)	0.042

Abbreviations: HR, hazard ratio; CI, confidential interval; AFP - alpha fetoprotein, TNM - tumor, node, metastasis; ALT, alanine aminotransferase. BCLC, Barcelona Clinic Liver Cancer. *P-Value<0.05 were considered statistically significant.

**Table 3:** Correlation between SNRPD1 expression and clinical outcomes in HCC patients (n=154).

Characteristics		N	SNRPD1 level		X^2^	*P-Value
			high(n)	low(n)		
Age (year)	>55	101	55	46	3.889	0.049
	<=55	53	20	33		
Gender	Male	134	66	68	0.126	0.723
	Female	20	9	11		
Tumor size (cm)	>5cm	82	37	45	0.889	0.343
	<=5cm	72	38	34		
TNM staging	I/II	103	39	57	6.655	0.010
	III	51	36	22		
Serum AFP level	>400ng/ml	81	50	31	11.607	0.001
	<=400ng/ml	73	25	48		
Tumor location	Left	52	21	31	2.174	0.140
	Right	102	54	48		
Tumor differentiation	Low	20	14	6	12.472	0.002
	Median	98	52	46		
	High	36	9	27		
Vascular invasion	Yes	73	44	29	7.440	0.006
	No	81	31	50		
Tumor encapsulation	Yes	104	50	54	0.050	0.823
	No	50	25	25		
HBV DNA load	>10^4^	72	38	33	1.225	0.268
	<=10^4^	82	37	46		
Recurrence	Yes	78	44	34	4.393	0.036
	No	76	31	45		
Survival	Alive	94	39	55	5.023	0.025
	Dead	60	36	24		

**Table 4:** Univariate and Multivariate Cox Regression analysis of overall survival and Recurrence-free survival in HCC patients (n=154).

Variables		Overall survival	*P-Value	Recurrence-free survival	*P-Value
		HR (95%CI)		HR (95%CI)	
Univariate analysis					
Age (year)	>55 vs. <=55	0.825(0.490-1.389)	0.470	0.732(0.463-1.158)	0.183
Gender	Male vs. female	0.779(0.335-1.810)	0.561	1.258(0.665-2.381)	0.480
Tumor size (cm)	>5 vs. <=5	1.391(0.705-2.744)	0.341	0.978(0.616-1.555)	0.926
TNM staging	I/II vs. III	1.963(1.144-3.367)	0.014	1.677(1.060-2.653)	0.027
Serum AFP level	>400 vs <=400	1.126(0.678-1.868)	0.647	1.246(0.799-1.943)	0.332
Tumor location	Left vs. right	0.757(0.450-1.275)	0.296	1.175(0.727-1.901)	0.510
Tumor differentiation	Hihg vs. median/low	1.613(0.971-2.679)	0.065	1.803(1.010-3.220)	0.046
Vascular invasion	Yes vs. no	1.747(1.045-2.921)	0.033	2.075(1.315-3.275)	0.002
Tumor encapsulation	Yes vs. no	0.788(0.466-1.334)	0.376	0.259(0.164-0.488)	<0.001
HBV DNA load	>10^4^ vs <=10^4^	1.395(0.840-2.314)	0.198	0.984(0.630-1.538)	0.944
SNRPD1	High vs. low	1.897(1.130-3.182)	0.015	1.642(1.049-2.571)	0.030
Multivariate analysis					
TNM staging	I/II vs. III	1.997(1.136-3.511)	0.016	1.682(1.049-2.699)	0.031
Tumor differentiation	Hihg vs. median/low	1.070(0.519-2.206)	0.854	1.166(0.624-2.180)	0.631
Vascular invasion	Yes vs. no	1.257(0.713-2.216)	0.430	1.861(1.122-3.087)	0.016
Tumor encapsulation	Yes vs. no			0.209(0.129-0.338)	<0.001
SNRPD1	High vs. low	1.890(1.098-3.255)	0.022	1.735(1.070-2.813)	0.026

Abbreviations: HR, hazard ratio; CI, confidential interval; AFP - alpha fetoprotein, TNM - tumor, node, metastasis. *P-Value<0.05 were considered statistically significant.

**Table 5:** The main GO and KEGG pathway enrichment analysis for 81 co-expressed genes.

Category	ID Term	Term	Count	*P-Value	Benjamini	FDR
GOTERM_Biological Process	GO:0051301	Cell division	21	6.60E-17	3.40E-14	3.25E-14
GOTERM_Biological Process	GO:0051170	Nuclear import	8	9.85E-13	2.54E-10	2.43E-10
GOTERM_Biological Process	GO:0000398	mRNA splicing, via spliceosome	14	2.31E-11	3.97E-09	3.80E-09
GOTERM_Biological Process	GO:0007067	Mitotic nuclear division	13	1.30E-09	1.67E-07	1.60E-07
GOTERM_Biological Process	GO:0000387	Spliceosomal snRNP assembly	7	2.65E-09	2.74E-07	2.61E-07
GOTERM_Biological Process	GO:0006364	rRNA processing	9	6.04E-06	4.45E-04	4.25E-04
GOTERM_Biological Process	GO:0008334	Histone mRNA metabolic process	4	1.98E-05	1.28E-03	1.22E-03
GOTERM_Biological Process	GO:0006369	Termination of RNA polymerase II transcription	5	2.11E-04	7.69E-03	7.34E-03
GOTERM_Biological Process	GO:0000245	Spliceosomal complex assembly	4	2.23E-04	7.69E-03	7.34E-03
GOTERM_Biological Process	GO:0008283	Cell proliferation	9	2.68E-04	8.64E-03	8.25E-03
GOTERM_Biological Process	GO:0008380	RNA splicing	6	9.89E-04	2.55E-02	2.44E-02
GOTERM_Biological Process	GO:0006281	DNA repair	6	4.49E-03	9.26E-02	8.85E-02
GOTERM_Biological Process	GO:0051726	Regulation of cell cycle	4	1.93E-02	2.77E-01	2.65E-01
KEGG_PATHWAY	hsa03040	Spliceosome	12	2.87E-09	1.95E-07	1.95E-07
KEGG_PATHWAY	hsa03010	Ribosome	6	3.46E-03	1.18E-01	1.18E-01
KEGG_PATHWAY	hsa04110	Cell cycle	5	1.40E-02	3.17E-01	3.17E-01

*P-Value<0.05 were considered statistically significant.

**Table 6:** The main enriched KEGG pathways of SNRPD1 High Expression in TCGA database.

KEGG enrichment signal pathway	ES	NES	NOM p-value	FDR q-value
KEGG_RIBOSOME	0.848	1.788	0.016	0.086
KEGG_BASE_EXCISION_REPAIR	0.627	1.857	0.004	0.083
KEGG_DNA_REPLICATION	0.763	1.839	0.008	0.077
KEGG_RNA_DEGRADATION	0.532	1.817	0.004	0.076
KEGG_RNA_POLYMERASE	0.603	1.785	0.008	0.067
KEGG_CELL_CYCLE	0.560	1.775	0.014	0.066
KEGG_MISMATCH_REPAIR	0.665	1.718	0.028	0.095
KEGG_NUCLEOTIDE_EXCISION_REPAIR	0.516	1.607	0.037	0.193
KEGG_HOMOLOGOUS_RECOMBINATION	0.658	1.729	0.019	0.095
KEGG_BLADDER_CANCER	0.496	1.639	0.010	0.164

*P-Value<0.05 were considered statistically significant.

## Abbreviations

SNRPD1: Small nuclear ribonucleoprotein Sm D1
HCC: hepatocellular carcinoma
IHC: immunohistochemistry
GSEA: Gene set enrichment analysis
HBV: hepatitis B virus
HCV: hepatitis C virus
AFP: Serum alpha-fetoprotein
GEO: Gene Expression Omnibus
GO: gene ontology
KEGG: The Kyoto Encyclopedia of Genes and Genomes
GEPIA: Gene Expression Profiling Interactive Analysis
TCGA: The Cancer Genome Atlas
CCLE: The Cancer Cell Line Encyclopedia project
OS: overall survival
RFS: recurrence-free survival
DAVID: Database for Annotation, Visualization and Integrated Discovery
PPI: protein-protein interaction
qRT-PCR: Quantitative polymerase chain reaction
TEM: transmission electron microscopy
LC3: Protein 1 Light Chain 3

## References

[1] Yang JD, Hainaut P, Gores GJ, Amadou A, Plymoth A, Roberts LR (2019). A global view of hepatocellular carcinoma: trends, risk, prevention and management. Nat Rev Gastroenterol Hepatol.

[2] Kulik L, El-Serag HB (2019). Epidemiology and Management of Hepatocellular Carcinoma. Gastroenterology.

[3] Quidville V, Alsafadi S, Goubar A, Commo F, Scott V, Pioche-Durieu C (2013). Targeting the deregulated spliceosome core machinery in cancer cells triggers mTOR blockade and autophagy. Cancer Res.

[4] Will CL, Luhrmann R (2011). Spliceosome structure and function. Cold Spring Harb Perspect Biol.

[5] Wan R, Bai R, Shi Y (2019). Molecular choreography of pre-mRNA splicing by the spliceosome. Curr Opin Struct Biol.

[6] Salgado-Garrido J, Bragado-Nilsson E, Kandels-Lewis S, Seraphin B (1999). Sm and Sm-like proteins assemble in two related complexes of deep evolutionary origin. EMBO J.

[7] Hermann H, Fabrizio P, Raker VA, Foulaki K, Hornig H, Brahms H (1995). snRNP Sm proteins share two evolutionarily conserved sequence motifs which are involved in Sm protein-protein interactions. EMBO J.

[8] Dvinge H, Guenthoer J, Porter PL, Bradley RK (2019). RNA components of the spliceosome regulate tissue- and cancer-specific alternative splicing. Genome Res.

[9] Engreitz JM, Haines JE, Perez EM, Munson G, Chen J, Kane M (2016). Local regulation of gene expression by lncRNA promoters, transcription and splicing. Nature.

[10] Lee SC, Abdel-Wahab O (2016). Therapeutic targeting of splicing in cancer. Nat Med.

[11] Sveen A, Kilpinen S, Ruusulehto A, Lothe RA, Skotheim RI (2016). Aberrant RNA splicing in cancer; expression changes and driver mutations of splicing factor genes. Oncogene.

[12] Pellagatti A, Armstrong RN, Steeples V, Sharma E, Repapi E, Singh S (2018). Impact of spliceosome mutations on RNA splicing in myelodysplasia: dysregulated genes/pathways and clinical associations. Blood.

[13] Zhan YT, Li L, Zeng TT, Zhou NN, Guan XY, Li Y (2020). SNRPB-mediated RNA splicing drives tumor cell proliferation and stemness in hepatocellular carcinoma. Aging (Albany NY).

[14] Jia D, Wei L, Guo W, Zha R, Bao M, Chen Z (2011). Genome-wide copy number analyses identified novel cancer genes in hepatocellular carcinoma. Hepatology.

[15] Liu N, Wu Z, Chen A, Wang Y, Cai D, Zheng J (2019). SNRPB promotes the tumorigenic potential of NSCLC in part by regulating RAB26. Cell Death Dis.

[16] Zhu L, Zhang X, Sun Z (2020). SNRPB promotes cervical cancer progression through repressing p53 expression. Biomed Pharmacother.

[17] Li Y, Ren Z, Peng Y, Li K, Wang X, Huang G (2019). Classification of glioma based on prognostic alternative splicing. BMC Med Genomics.

[18] Hu C, Li M, Liu J, Qian J, Xu D, Zhang S (2017). Anti-SmD1 antibodies are associated with renal disorder, seizures, and pulmonary arterial hypertension in Chinese patients with active SLE. Sci Rep.

[19] Kim YD, Lee J, Kim HS, Lee MO, Son MY, Yoo CH (2017). The unique spliceosome signature of human pluripotent stem cells is mediated by SNRPA1, SNRPD1, and PNN. Stem Cell Res.

[20] Batra R, Harder N, Gogolin S, Diessl N, Soons Z, Jager-Schmidt C (2012). Time-lapse imaging of neuroblastoma cells to determine cell fate upon gene knockdown. PLoS One.

[21] Ogata H, Goto S, Sato K, Fujibuchi W, Bono H, Kanehisa M (1999). KEGG: Kyoto Encyclopedia of Genes and Genomes. Nucleic Acids Res.

[22] Tang Z, Li C, Kang B, Gao G, Li C, Zhang Z (2017). GEPIA: a web server for cancer and normal gene expression profiling and interactive analyses. Nucleic Acids Res.

[23] Uhlen M, Fagerberg L, Hallstrom BM, Lindskog C, Oksvold P, Mardinoglu A (2015). Proteomics. Tissue-based map of the human proteome. Science.

[24] Gao J, Aksoy BA, Dogrusoz U, Dresdner G, Gross B, Sumer SO (2013). Integrative analysis of complex cancer genomics and clinical profiles using the cBioPortal. Sci Signal.

[25] Edgar R, Domrachev M, Lash AE (2002). Gene Expression Omnibus: NCBI gene expression and hybridization array data repository. Nucleic Acids Res.

[26] Ke R, Lv L, Li J, Zhang X, Yang F, Zhang K (2018). Prognostic value of heterogeneous ribonucleoprotein A1 expression and inflammatory indicators for patients with surgically resected hepatocellular carcinoma: Perspectives from a high occurrence area of hepatocellular carcinoma in China. Oncol Lett.

[27] Zhao S, Wang M, Yang Z, Tan K, Zheng D, Du X (2020). Comparison between Child-Pugh score and Albumin-Bilirubin grade in the prognosis of patients with HCC after liver resection using time-dependent ROC. Ann Transl Med.

[28] Kee KM, Wang JH, Lee CM, Chen CL, Changchien CS, Hu TH (2007). Validation of clinical AJCC/UICC TNM staging system for hepatocellular carcinoma: analysis of 5,613 cases from a medical center in southern Taiwan. Int J Cancer.

[29] Vasaikar SV, Straub P, Wang J, Zhang B (2018). LinkedOmics: analyzing multi-omics data within and across 32 cancer types. Nucleic Acids Res.

[30] Dennis G Jr, Sherman BT, Hosack DA, Yang J, Gao W, Lane HC (2003). DAVID: Database for Annotation, Visualization, and Integrated Discovery. Genome Biol.

[31] Franceschini A, Szklarczyk D, Frankild S, Kuhn M, Simonovic M, Roth A (2013). STRING v9.1: protein-protein interaction networks, with increased coverage and integration. Nucleic Acids Res.

[32] Goldman MJ, Craft B, Hastie M, Repecka K, McDade F, Kamath A (2020). Visualizing and interpreting cancer genomics data via the Xena platform. Nat Biotechnol.

[33] Subramanian A, Tamayo P, Mootha VK, Mukherjee S, Ebert BL, Gillette MA (2005). Gene set enrichment analysis: a knowledge-based approach for interpreting genome-wide expression profiles. Proc Natl Acad Sci U S A.

[34] Barth S, Glick D, Macleod KF (2010). Autophagy: assays and artifacts. J Pathol.

[35] Jiang P, Mizushima N (2015). LC3- and p62-based biochemical methods for the analysis of autophagy progression in mammalian cells. Methods.

[36] Wang E, Aifantis I (2020). RNA Splicing and Cancer. Trends Cancer.

[37] Urbanski LM, Leclair N, Anczukow O (2018). Alternative-splicing defects in cancer: Splicing regulators and their downstream targets, guiding the way to novel cancer therapeutics. Wiley Interdiscip Rev RNA.

[38] Niu HT, Yang CM, Chen B, Dong Q (2011). Biomarker research and some deduction in superficial bladder cancer cells combined with corresponding stroma. Cancer Biomark.

[39] Yi M, Li T, Qin S, Yu S, Chu Q, Li A (2020). Identifying Tumorigenesis and Prognosis-Related Genes of Lung Adenocarcinoma: Based on Weighted Gene Coexpression Network Analysis. Biomed Res Int.

[40] Dai X, Yu L, Chen X, Zhang J (2021). SNRPD1 confers diagnostic and therapeutic values on breast cancers through cell cycle regulation. Cancer Cell Int.

[41] Yoshida K, Sanada M, Shiraishi Y, Nowak D, Nagata Y, Yamamoto R (2011). Frequent pathway mutations of splicing machinery in myelodysplasia. Nature.

[42] Sampath J, Long PR, Shepard RL, Xia X, Devanarayan V, Sandusky GE (2003). Human SPF45, a splicing factor, has limited expression in normal tissues, is overexpressed in many tumors, and can confer a multidrug-resistant phenotype to cells. Am J Pathol.

[43] Lennerz V, Fatho M, Gentilini C, Frye RA, Lifke A, Ferel D (2005). The response of autologous T cells to a human melanoma is dominated by mutated neoantigens. Proc Natl Acad Sci U S A.

[44] Mishra S, Yadav T, Rani V (2016). Exploring miRNA based approaches in cancer diagnostics and therapeutics. Crit Rev Oncol Hematol.

[45] Ali Syeda Z, Langden SSS, Munkhzul C, Lee M, Song SJ (2020). Regulatory Mechanism of MicroRNA Expression in Cancer. Int J Mol Sci.

[46] Ke RS, Zhang K, Lv LZ, Dong YP, Pan F, Yang F (2019). Prognostic value and oncogene function of heterogeneous nuclear ribonucleoprotein A1 overexpression in HBV-related hepatocellular carcinoma. Int J Biol Macromol.

[47] Zhou HC, Fang JH, Shang LR, Zhang ZJ, Sang Y, Xu L (2016). MicroRNAs miR-125b and miR-100 suppress metastasis of hepatocellular carcinoma by disrupting the formation of vessels that encapsulate tumour clusters. J Pathol.

[48] Ge YY, Shi Q, Zheng ZY, Gong J, Zeng C, Yang J (2014). MicroRNA-100 promotes the autophagy of hepatocellular carcinoma cells by inhibiting the expression of mTOR and IGF-1R. Oncotarget.

[49] Ye Y, Li SL, Wang JJ (2020). miR-100-5p Downregulates mTOR to Suppress the Proliferation, Migration, and Invasion of Prostate Cancer Cells. Front Oncol.

[50] Yu Z, Li N, Jiang K, Zhang N, Yao LL (2020). MiR-100 up-regulation enhanced cell autophagy and apoptosis induced by cisplatin in osteosarcoma by targeting mTOR. Eur Rev Med Pharmacol Sci.

[51] Lin L, Huang Y, Zhuang W, Lin P, Ma X (2020). miR-100 inhibits cell proliferation in mantle cell lymphoma by targeting mTOR. Exp Hematol Oncol.

[52] Ge YY, Shi Q, Zheng ZY, Gong J, Zeng C, Yang J (2014). MicroRNA-100 promotes the autophagy of hepatocellular carcinoma cells by inhibiting the expression of mTOR and IGF-1R. Oncotarget.

